# Estimating indoor air temperature by obtaining outdoor building window surface temperature using infrared technology: An exploratory approach

**DOI:** 10.1016/j.buildenv.2024.111218

**Published:** 2024-03

**Authors:** Tingting Jiang, Fulin Hao, Xiaomeng Chen, Ziwei Zou, Shu Zheng, Yabin Liu, Shan Xu, Haiquan Yin, Xudong Yang

**Affiliations:** a Department of Building Science, School of Architecture, Tsinghua University, Beijing, 100084, China; b College of Information and Electrical Engineering, China Agricultural University, Beijing, 100084, China; c WISDRI Engineering & Research Incorporation Limited, Wuhan, 430223, China; d Beijing District Heating Group, Beijing, 100022, China; e Shanxi Research Institute for Clean Energy, Tsinghua University, Taiyuan, 030032, China

**Keywords:** Infrared thermography, Indoor air temperature, Window surface temperature, Numerical model, Heat transfer process

## Abstract

The determination of the indoor air temperature is necessary for evaluating human comfort, health, and living conditions. Existing measuring methods require entering a room, which can disturb the daily lives of residents and consume large amounts of manpower, material, and financial resources. To overcome these obstacles, an exploratory approach was proposed in this study to estimate the indoor air temperature by obtaining the outdoor building window surface temperature without intrusion using infrared technology. A numerical model was established to describe the heat transfer process between the indoor and outdoor air via window glass. Experiments were conducted in a test room to capture infrared images of the exterior window and measure indoor air temperatures and window surface temperatures under different modes. The estimated indoor air temperatures were compared with the experimental data. The effects of window property parameters and ambient parameters on indoor air temperature estimation were analyzed. Results show that the deviations of the indoor air temperature between estimated and measured values in heating, natural ventilation, and cooling modes varied from –0.7 °C to 0.6 °C, –1.1 °C–0.7 °C, and –0.1 °C–1.3 °C, respectively. Based on the sensitivity analysis, the outer surface temperature of the window outer layer was crucial for estimating the accuracy of the indoor air temperature in practical applications. The proposed exploratory approach provides a potential means for remotely obtaining indoor air temperatures using infrared technology.

## 1 Introduction

In modern society, people spend most of their time indoors. The indoor environment directly affects the thermal comfort and work efficiency of occupants and is closely related to their psychological and physical health [1,2]. Extremely high and low indoor temperatures can pose health risks, especially for the elderly, children, and patients with chronic diseases [3,4]. Jevons et al. [5] evaluated the impact of indoor air temperature thresholds on human health in English homes in winter, and the results showed that the physical health of the general population was threatened around 18.0 °C. In addition, a low-indoor-temperature environment had a significant impact on the health of the elderly because of their sometimes weak perceptual abilities. Tham et al. [6] identified evidence of the direct and indirect effects of high indoor temperatures on human health and suggested that an indoor air temperature setting of 26 °C was the most suitable for high-risk groups, such as patients with diabetes, schizophrenia, and dementia. The effects of crucial microclimatic parameters on human health, work, cognitive performance, and infection risk were integrated by Wolkoff et al. [7], who demonstrated that low temperatures increased the risk of cardiovascular and respiratory diseases, while high temperatures increased the risk of acute non-specific symptoms. Additionally, both high and low temperatures can lead to decreased learning performance and efficiency. Therefore, it is crucial to determine the indoor air temperature accurately to evaluate human health [8].

### 1.1 Literature review

Traditional methods for measuring indoor air temperatures include using wireless temperature sensors to remotely obtain continuous data or real-time temperature sensors to collect single-point data in households, distributing survey questionnaires, and directly inquiring about users’ feelings [9,10]. Miguel-Bellod et al. [11] adopted portable temperature data loggers to monitor the temperatures of living rooms and main bedrooms at a height of 1.5 m in 112 multifamily dwellings, and investigated the main building determinants of indoor temperature variation. Their results showed that families with financial constraints generally lived in rooms with lower indoor air temperatures. Wang et al. [12] distributed screening questionnaires to children’s parents or guardians to assess their health, indoor air quality, and living conditions in different regions, and the results showed that indoor air temperature was remarkably correlated with the prevalence of the common cold. A temperature monitoring survey of social housing dwellings in Central England was conducted by Morey et al. [13], and their analysis demonstrated that vulnerable groups could not tolerate or adapt to high indoor air temperatures during the summer. Bo et al. [14] arranged intelligent sensors on a wall to measure the indoor operative temperature in Yichun, Harbin, Shenyang, and Dalian and confirmed the severity of indoor overheating. However, the existing methods of obtaining the indoor air temperature not only require large amounts of manpower, material, and financial resources, but also face difficulties in entering households, communication, and maintenance [15]. In addition, they have the disadvantages of overly one-sided measurement data and limited sample size of users [16].

In recent years, infrared thermography has attracted great interest of researchers, which is a non-destructive imaging technique used to display the infrared radiation emitted by different element surfaces [17, 18]. It adopts optoelectronic technology to detect infrared specific band signals of object thermal radiation and converts the signal into images and graphics that can be distinguished by human vision [19,20]. Currently, infrared thermography has a wide range of applications in building diagnosis, including determination of the overall thermal conductivity of exterior walls and windows and detection of wall leakage, moisture, and thermal bridge defects in building envelopes [21, 22]. Fokaides et al. [23] proposed a method for identifying the *U*-Value of building envelopes using infrared thermography, and the obtained results were validated using measured data from a thermohygrometer in summer and winter. This study pointed out that the deviations between the notional and measured *U*-Values were acceptable and within the range of 10% – 20%. Al-Kassir et al. [24] used an infrared thermographic technique to evaluate the thermal patterns of large walls, barriers with constructive elements, pipes and conduits of HVAC systems, cables, wires, and contact breakers of electrical installations. The results demonstrated the relationship between the water evaporation and porosity in practical applications. An experimental campaign was conducted by Lerma et al. [25] to evaluate the application potential of active infrared thermography, and they effectively proved that the method of active infrared thermography combined with pressure differences could effectively detect air infiltration. Based on in-situ inspections and numerical simulations, Edis et al. [26] demonstrated that passive thermography with solar heat gain played a prominent role in detecting moisture content changes in adhered ceramic claddings. In addition, a few researchers have used infrared thermography to measure indoor air temperatures. For example, Fokaides et al. [27] developed a novel technique for mock-target infrared thermography to determine the indoor air temperature in a laboratory environment, optimize target material selection, and assess prerequisite conditions necessary to attain the required thermal equilibrium between the mock target and indoor environment. To achieve precise quantitative measurements of building interiors and structures through infrared thermography, Porras-Amores et al. [28] proposed a low-cost, portable measuring screen system, citing its reliability and high accuracy for avoiding emissivity, focus, and reflected temperature. Lee and Jo [29] used a pan-tilt infrared camera to remotely measure the interior surface temperature in indoor spaces and verified their results using experimental data. Results showed that the estimated MRT values aligned with the measured values. Both numerical analysis and laboratory experiments were performed by Georgiou et al. [30] to investigate the precision of IR-obtained transient data, wherein they concluded that mock-target IR thermography was capable of accurately predicting the transient air temperature, with an average temperature deviation of 0.4%.

### 1.2 Research gap and scope of study

In previous studies, the use of infrared thermography to determine the indoor air temperature generally occurred inside rooms, which can disturb the daily lives of occupants. Besides, these methods consume considerable time and labor to arrange the temperature sensor in every household in various regions and even cities [31]. To overcome these limitations, Chen et al. [32] adopted an infrared camera to detect the indoor air temperature remotely from outside of the building when the window was opened and confirmed the feasibility and accuracy of this method through experimental data. As the windows are not always opened in residential buildings, how can infrared cameras be used to obtain the indoor air temperature when the windows are closed? Therefore, based on the remote sensing characteristics of infrared thermography, an exploratory approach is proposed here to estimate the indoor air temperature by obtaining the building window surface temperature from outside using infrared technology. The following research questions are addressed to clarify the applicability of this method.

(1) How can we establish a relationship between the window surface temperature and indoor air temperature to achieve non-contact measurement?
(2) To what extent does the infrared temperature of the window surface reflect the indoor air temperature?
(3) What is the feasibility and accuracy of this method?

In this study, a numerical model was established to describe the heat transfer process between the indoor and outdoor air through window glass. Experiments were conducted in a laboratory, and the reliability of the numerical model was validated using measured data. Then, the accuracy of estimating the indoor air temperature using the above method and window opening gap method was compared. Subsequently, the effects of window property parameters and ambient parameters on indoor air temperature estimation were investigated. Finally, the correlation degrees of these influencing factors on the prediction of the indoor air temperature were derived.

## 2 Methodology

### 2.1 Physical model

**Fig. 1:**
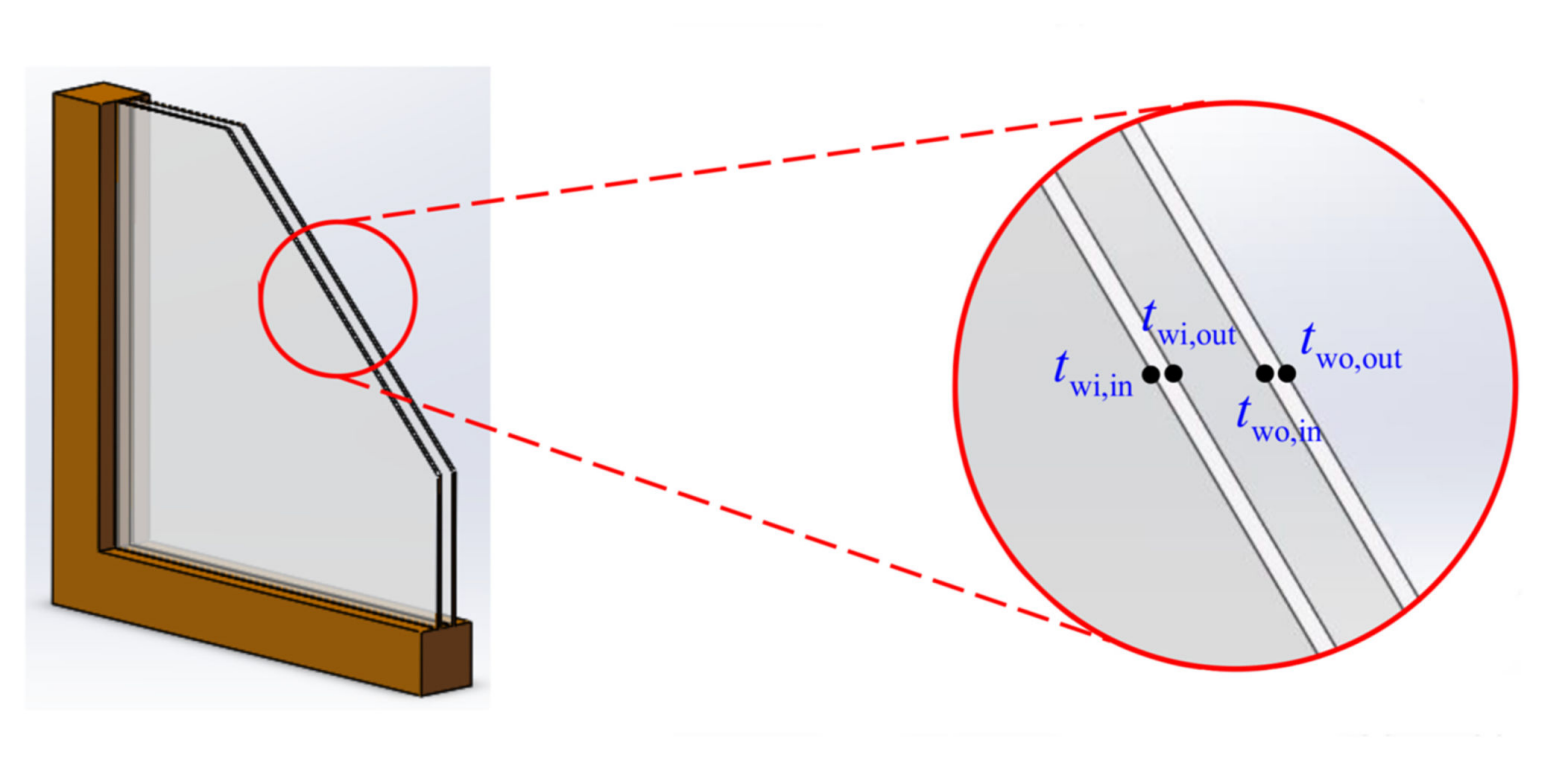
The schematic of the window structure.

Fig. 1 shows a schematic of the window structure, which consists of a window frame, inner window glass, hollow sandwich, and outer window glass. The inner and outer glasses are embedded in a window frame. During the heat transfer process, the heat from the indoor air and interior surface of walls is transferred to the inner surface of the window inner layer by natural convection in infinite space and radiation, and then delivered to the outer surface of the window inner layer by heat conduction. Subsequently, the outer surface of the window inner layer transfers heat to the inner surface of the window outer layer via natural convection in enclosed space and radiation, and then to the outer surface of the window outer layer via heat conduction. Finally, the heat from the outer surface of the window outer layer is delivered to the outdoor air through convection and radiation [33].

### 2.2 Numerical model

A numerical model is established to describe the heat transfer characteristics between the indoor and outdoor air through window glass, and the following assumptions are made.

(1) The heat transfer process between the indoor and outdoor air through window glass is assumed to be one-dimensional and in a steady state.
(2) The heat conduction losses that occur in the axial direction of the inner surface of the window inner layer, outer surface of the window inner layer, inner surface of the window outer layer, and outer surface of the window outer layer are ignored.
(3) The window glass is not directly exposed to sunlight, thus the effect of solar radiation is ignored.

The input parameters of the numerical model include the window height, hollow sandwich thickness, thermal conductivity and thickness of the window glass, glass surface emissivity, the outer surface temperature of the window outer layer, outdoor air temperature, indoor and outdoor wind velocity. The temperature of the outer surface of the window outer layer is obtained by infrared data, and other parameters are derived from experimental values. The temperatures of the indoor air, inner surface of the window inner layer, outer surface of the window inner layer, inner surface of the window outer layer, outer surface of the window outer layer, and outdoor air are defined as *t*_a_, *t*_wi,in_, *t*_wi,out_, *t*_wo,in_, *t*_wo,out_, *t*_e_, respectively. The energy balance equations are as follows:

For the inner surface of the window inner layer:

(1) $$Q_{1}-Q_{01}=0$$

(2) $$Q_{1}=h_{\text{a}-\text{wi,in}}\cdot A\cdot \left(t_{\text{a}}-t_{\text{wi},in}\right)+\sigma \cdot \varepsilon \cdot A\cdot \left(t_{\text{a}}^{4}-t_{\text{wi,in}}^{4}\right)$$

(3) $$Q_{01}=\frac{\lambda_{\text{w}}}{\delta_{\text{w}}}\cdot A\cdot \left(t_{\text{wi,in}}-t_{\text{wi,out}}\right)$$

where *Q*_1_ is the heat exchange capacity between the inner surface of the window inner layer and indoor air, W; *Q*_01_ is the heat exchange capacity between the inner surface of the window inner layer and the outer surface of the window inner layer, W; *h*_a-wi,in_ is the convective heat transfer coefficient between the inner surface of the window inner layer and indoor air, W⋅m^−2^⋅K^−1^; *A* is the surface area of the control volume, m^2^; σ is the Stefan-Boltzmann constant, 5.67 × 10^−8^ W m^−2^ K^−4^; *ε* is the emissivity of the glass surface; *λ*_w_ is the thermal conductivity of the window glass, W⋅m^−1^⋅K^−1^; and *δ*_w_ is the thickness of the window glass, m.

For the outer surface of the window inner layer:

(4) $$Q_{01}-Q_{0}=0$$

(5) $$Q_{0}=h_{\text{wi},\text{out}-\text{wo},\text{in}}\cdot A\cdot \left(t_{\text{wi},\text{out}}-t_{\text{wo,in}}\right)+\sigma \cdot \varepsilon_{\text{b}}\cdot A\cdot \left(t_{\text{wi},\text{out}}^{4}-t_{\text{wo,in}}^{4}\right)$$

where *Q*_0_ is the heat exchange capacity between the outer surface of the window inner layer and the inner surface of the window outer layer, W; *h*_wi,out-wo,in_ is the convective heat transfer coefficient between the outer surface of the window inner layer and the inner surface of the window outer layer, W⋅m^−2^⋅K^−1^; and ε_b_ is the intersurface emissivity of the glass sandwich.

For the inner surface of the window outer layer:

(6) $$Q_{0}-Q_{02}=0$$

(7) $$Q_{02}=\frac{\lambda_{\text{w}}}{\delta_{\text{w}}}\cdot A\cdot \left(t_{\text{wo},\text{in}}-t_{\text{wo},\text{out}}\right)$$

where *Q*_02_ is the heat exchange capacity between the inner surface of the window outer layer and the outer surface of the window outer layer, W.

For the outer surface of the window outer layer:

(8) $$Q_{02}-Q_{2}=0$$

(9) $$Q_{2}=h_{\text{wo},\text{out}-\text{e}\;}\cdot A\cdot \left(t_{\text{wo},\text{out}}-t_{\text{e}}\right)+\sigma \cdot \varepsilon \cdot A\cdot \left(t_{\text{wo},\text{out}}^{4}-t_{\text{e}}^{4}\right)$$

where *Q*_2_ is the heat exchange capacity between the outer surface of the window outer layer and outdoor air, W; and *h*_wo,out-e_ is the convective heat transfer coefficient between the outer surface of the window outer layer and outdoor air, W⋅m^−2^⋅K^−1^.

When the heat transfer between the inner surface of the window inner layer and indoor air is natural convection, the convective heat transfer coefficient *h*_a-wi,in_ can be defined as [34,35]:

(10) $$h_{\text{a}-\text{wi,in}}=\frac{\lambda_{1}}{H}Nu_{\text{f}1}$$

(11) $$Nu_{\text{f}1}=0.59\cdot {\left(Gr_{\text{H}1}\cdot Pr_{1}\right)}^{1/4}\;10^{4}<Gr_{\text{H}1}\cdot Pr_{1}<10^{9}$$

(12) $$Nu_{\text{f}1}=0.1\cdot {\left(Gr_{\text{H}1}\cdot Pr_{1}\right)}^{1/3}\;10^{9}<Gr_{\text{H}1}\cdot Pr_{1}<10^{13}$$

(13) $$Gr_{\text{H}1}=\frac{g\alpha_{1}\text{Δ}t_{1}H^{3}}{\nu_{1}^{2}}$$

where *λ*_1_ is the thermal conductivity of the inner surface boundary layer of the window inner layer, W⋅m^−1^⋅K^−1^; *H* is the height of the window, m; *Nu*_f1_ is the Nusselt number of the convective heat transfer between the inner surface of the window inner layer and indoor air; *Gr*_H1_ is the Grashov number of the inner surface boundary layer of the window inner layer; *Pr*_1_ is the Prandtl number of the inner surface boundary layer of the window inner layer; *g* is the gravitational acceleration, m⋅s^−2^; *α*_1_ is the volume expansion coefficient of the inner surface boundary layer of the window inner layer, 1 K^−1^; Δ*t*_1_ is the temperature difference between the inner surface of the window inner layer and indoor air, °C; and *ν*_1_ is the kinematic viscosity of the inner surface boundary layer of the window inner layer, m^2^⋅s.

When the heat transfer between the inner surface of the window inner layer and indoor air is considered as forced convection, the convective heat transfer coefficient *h*_a-wi,in_ can be determined as [36]:

(14) $$h_{\text{a}-\text{w,in}}=3\cdot v_{\text{f},\text{a}}+2.8$$

where *v*_f,a_ is the indoor wind velocity, m⋅s^−1^.

The convective heat transfer coefficient between the outer surface of the window inner layer and the inner surface of the window outer layer *h*_wi,out-wo,in_ is expressed as [37,38]:

(15) $$h_{\text{a}-\text{wi,in}}=\frac{\lambda_{0}}{\delta_{0}}Nu_{\text{f}0}$$

(16) $$Nu_{\text{f}0}=1Gr_{\text{H}0}\leq 2000$$

(17) $$Nu_{\text{f}0}=0.197\cdot {\left(Gr_{\text{H}0}\cdot Pr_{0}\right)}^{1/4}{\left(\frac{\delta_{0}}{H}\right)}^{1/9}\;\text{if}\;\left\{\begin{array}{l} 6000<Gr_{\text{H}0}\cdot Pr_{0}<2\times 10^{5}\\ 11<H/\delta_{0}<42 \end{array}\right\}$$

(18) $$Nu_{\text{f}0}=0.073\cdot {\left(Gr_{\text{H}0}\cdot Pr_{0}\right)}^{1/3}{\left(\frac{\delta_{0}}{H}\right)}^{1/9}\;\text{if}\;\left\{\begin{array}{l} 2\times 10^{5}<Gr_{\text{H}0}\cdot Pr_{0}<1.1\times 10^{7}\\ 11\leq H/\delta_{0}\leq 42 \end{array}\right\}$$

(19) $$Gr_{\text{H}0}=\frac{g\alpha_{0}\text{Δ}t_{0}\delta_{0}^{3}}{\nu_{0}^{2}}$$

where *λ*_0_ is the thermal conductivity of the air in insulating glass, W⋅m^−1^⋅K^−1^; *δ*_0_ is the spacing of insulating glass, m; *Nu*_f0_ is the Nusselt number of the convective heat transfer between the outer surface of the window inner layer and the inner surface of the window outer layer; *Gr*_H0_ is the Grashov number of the air in insulating glass; *Pr*_0_ is the Prandtl number of the air in insulating glass; *α*_0_ is the volume expansion coefficient of the air in insulating glass, 1 K^−1^; Δ*t*_0_ is the temperature difference between the outer surface of the window inner layer and the inner surface of the window outer layer, °C; and *ν*_0_ is the kinematic viscosity of the air in insulating glass, m^2^⋅s.

When the heat transfer between the outer surface of the window outer layer and outdoor air is natural convection, the convective heat transfer coefficient *h*_wo,out-e_ can be calculated as follows [39]:

(20) $$h_{\text{wo},\text{out}-e\;}=\frac{\lambda_{2}}{H}Nu_{\text{f}2}$$

(21) $$Nu_{\text{f}2}=0.59\cdot {\left(Gr_{\text{H}2}\cdot Pr_{2}\right)}^{1/4}10^{4}<Gr_{\text{H}2}\cdot Pr_{2}<10^{9}$$

(22) $$Nu_{\text{f}2}=0.1\cdot {\left(Gr_{\text{H}2}\cdot Pr_{2}\right)}^{1/3}10^{9}<Gr_{\text{H}2}\cdot Pr_{2}<10^{13}$$

(23) $$Gr_{\text{H}2}=\frac{g\alpha_{2}\text{Δ}t_{2}H^{3}}{\nu_{2}^{2}}$$

where *λ*_2_ is the thermal conductivity of the outer surface boundary layer of the window outer layer, W⋅m^−1^⋅K^−1^; *Nu*_f2_ is the Nusselt number of the convective heat transfer between the outer surface of the window outer layer and outdoor air; *Gr*_H2_ is the Grashov number of the outer surface boundary layer of the window outer layer; *Pr*_2_ is the Prandtl number of the outer surface boundary layer of the window outer layer; *α*_2_ is the volume expansion coefficient of the outer surface boundary layer of the window outer layer, 1 K^−1^; Δ*t*_2_ is the temperature difference between the outer surface of the window outer layer and outdoor air, °C; and *ν*_2_ is the kinematic viscosity of the outer surface boundary layer of the window outer layer, m^2^⋅s.

When the outer surface of the window outer layer depends on forced convection to exchange heat with outdoor air, the convective heat transfer coefficient *h*_wo,out-e_ can be expressed as [40]:

(24) $$h_{\text{wo},\text{out}\;-\text{e}}=4+4\cdot v_{\text{f},\text{e}}$$

where *v*_f,e_ is the outdoor wind velocity, m⋅s^−1^.

The intersurface emissivity of the glass sandwich, *ε*_b_ is expressed as [41]:

(25) $$\varepsilon_{\text{b}}=\frac{1}{\frac{1}{\varepsilon }+\frac{1}{\varepsilon }-1}$$

### 2.3 Calculation algorithm

**Fig. 2:**
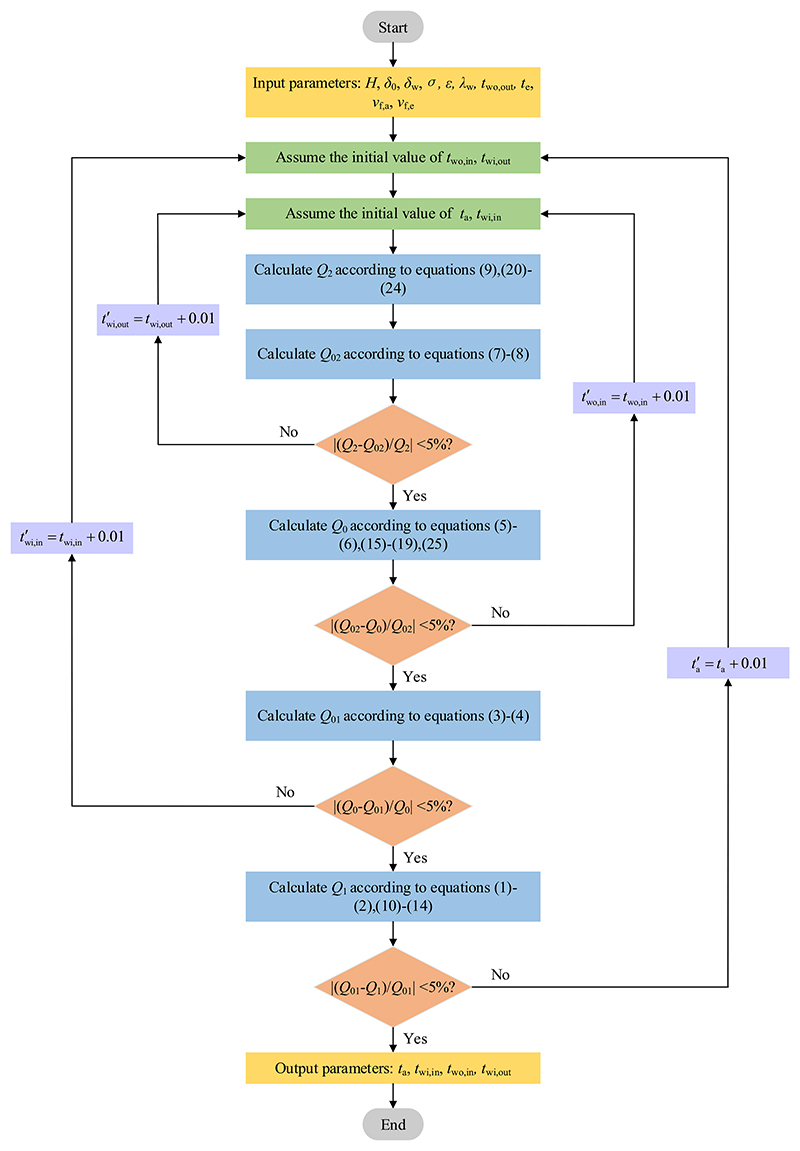
Flowchart of the calculation algorithm.

A flowchart of the calculation algorithm is presented in Fig. 2. The calculation steps are as follows: first, input geometrical and physical parameters of the window, *t*_wo,out_, *t*_e_, *v*_f,a_, *v*_f,e_, and assume the initial value of the *t*_a_, *t*_wi,in_, *t*_wi,out_, *t*_wo,in_, respectively. Second, calculate the heat exchange capacity between the outer surface of the window outer layer and outdoor air *Q*_2_, then calculate the heat exchange capacity between the inner surface of the window outer layer and the outer surface of the window outer layer *Q*_02_ using iteration method until the relative error between the calculated value and *Q*_2_ is less than 5%. Third, calculate the heat exchange capacity between the outer surface of the window inner layer and the inner surface of the window outer layer *Q*_0_ using iteration method until the convergence condition is satisfied. Fourth, based on the above calculation method, calculate the heat exchange capacity between the inner surface of the window inner layer and the outer surface of the window inner layer *Q*_01_ and the heat exchange capacity between the inner surface of the window inner layer and indoor air *Q*_1_. Finally, output all values of the *t*_a_, *t*_wi,in_, *t*_wi,out_, *t*_wo,in_, respectively.

## 3 Experiments

### 3.1 Test rig

**Fig. 3:**
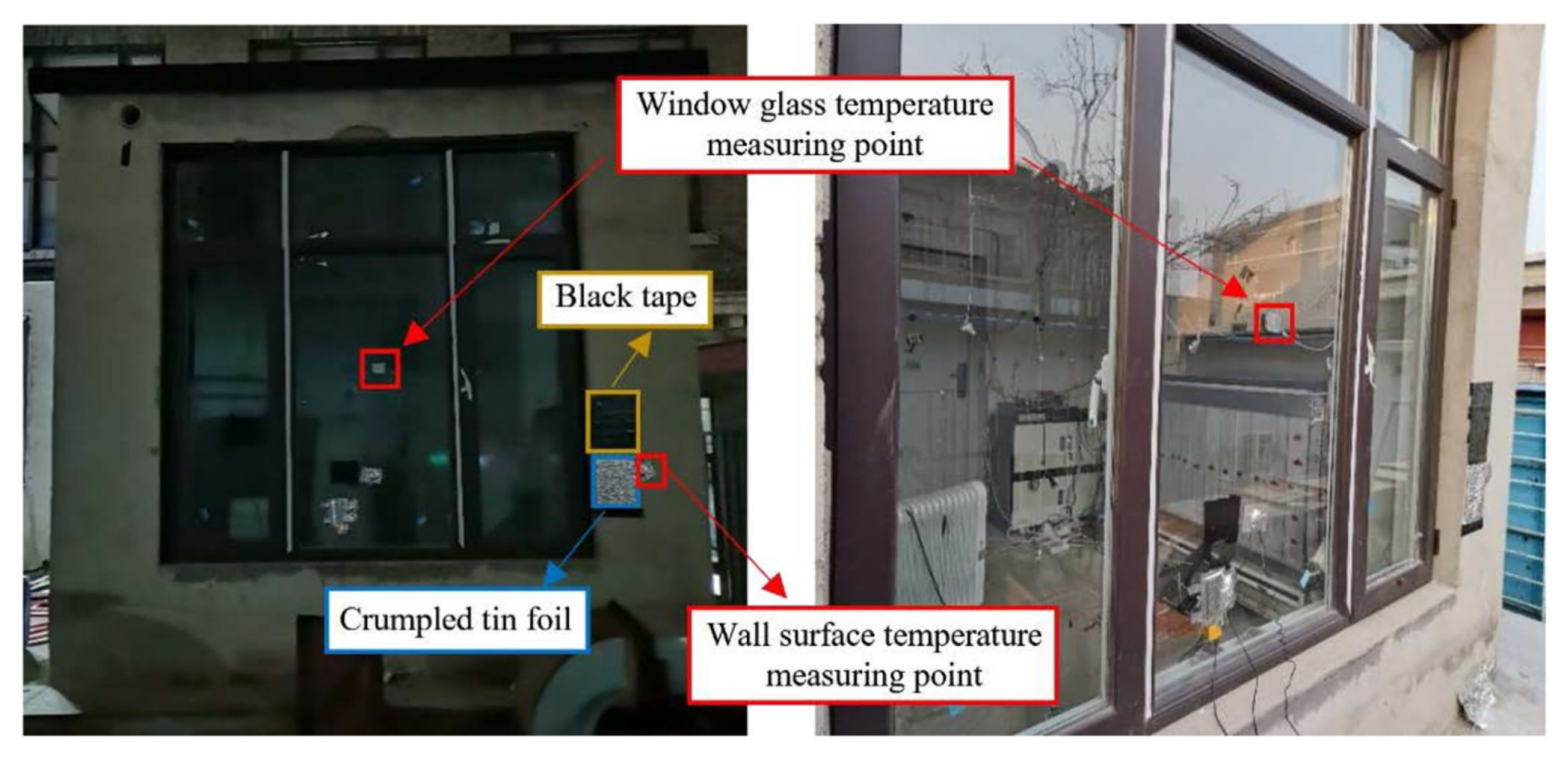
The picture of the test room.

To validate the precision of the established numerical model, experiments were conducted in a laboratory on the campus of the Tsinghua University in Beijing, China. A constructed test room was built out of 30-cm-thick concrete to simulate an indoor environment, with the dimensions of 2.7 m × 2.7 m × 2.7 m (L × W × H). As shown in Fig. 3, the exterior window of double-layer vacuum glass was embedded in the south wall of the test room. The thicknesses of glass and hollow sandwich were 3 mm and 12 mm, respectively. The emissivity of the glass surface was set according to Technical Specification for Application of Architectural Glass JGJ 113–2015 [42]. The crumpled piece of tin foil was fixed on the external wall to determine the reflected ambient temperature. To avoid the specular reflection of surrounding buildings, the black tape was used to correct image capture errors as detailed in Section 4. The emissivity of the black tape was set as 0.95 [43]. The thermal environment of the test room was maintained using a radiator and fan coil during the winter and summer, respectively. The radiator and fan coil used water as the working fluid, which originated from a thermostat water tank positioned in the plant room. In the experiments, an infrared camera (VarioCAM) was used to capture the exterior window images of the test room. To maintain a consistent horizontal view of the infrared camera lens and target window, the height of the infrared camera tripod was adjusted. The infrared camera was arranged 5.0 m away from the target window. To avoid the effect of solar radiation on infrared images, the experiments were carried out at night, with the outdoor wind speed of 0.12 m s^−1^.

During the experiments, the indoor thermal environment of the test room was controlled in heating, natural ventilation, and cooling modes. The conditions and environmental parameters of the experimental chamber are listed in Table 1. In heating mode, the radiator depended on natural convection and radiation to exchange heat with the indoor environment. In cooling mode, the fan coil sent cold air to the test room via forced convection. In natural ventilation mode, the radiator and fan coil were turned off. To ensure that the indoor air temperature was stable, infrared images were captured after the radiator or fan coil ran for 10 h. Infrared images were recorded at intervals of approximately 60 s.

### 3.2 Data collection

**Fig. 4:**
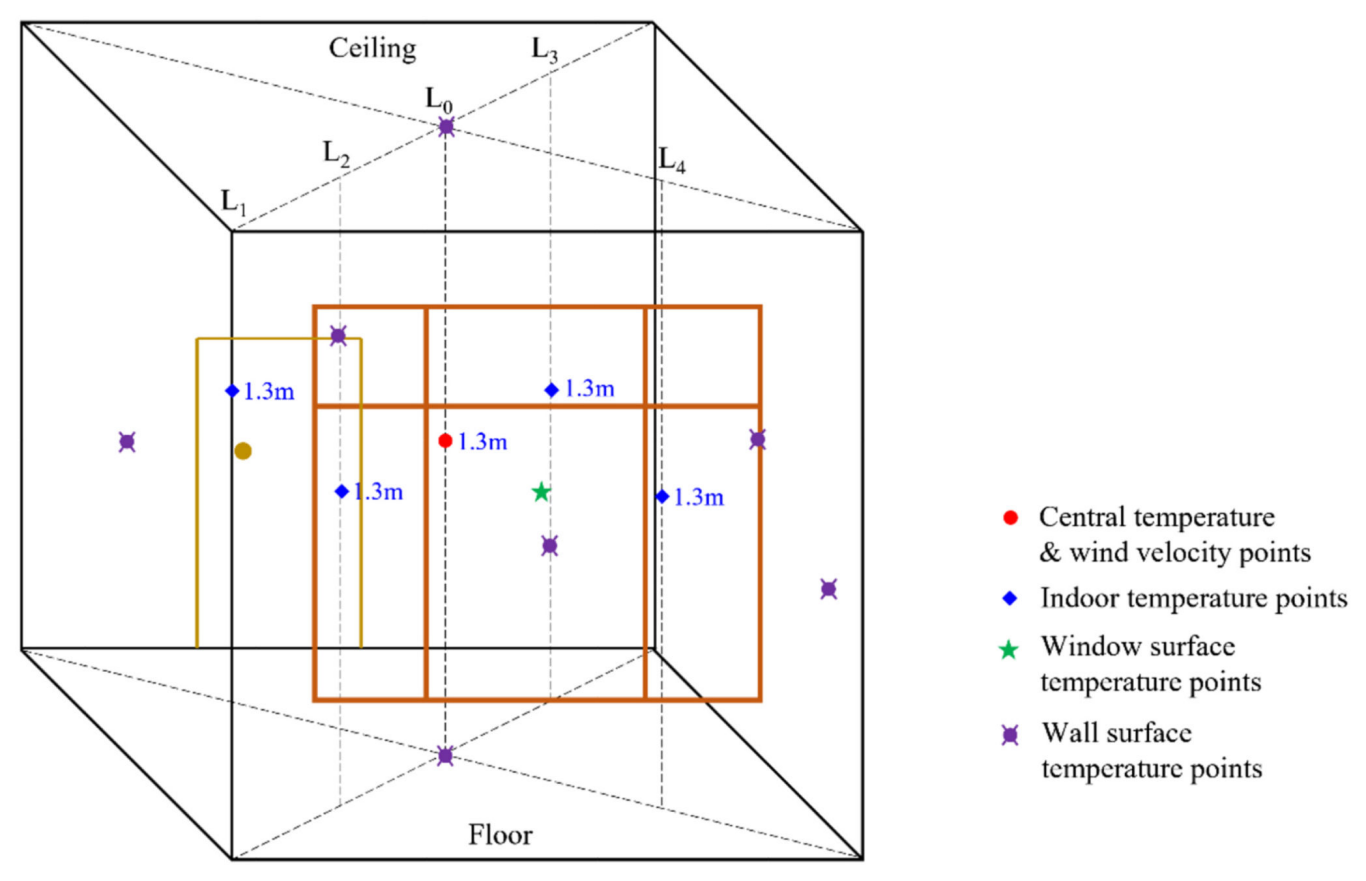
Parameter measurement in the test room.

As shown in Fig. 4, the orange outlines represent the window at the south wall and the yellow outlines represent the door of the test room. To monitor the indoor ambient parameters, six temperature points were arranged on the ceiling, floor, and four walls. The measured temperature at the indoor center point at a height of 1.3 m was used to represent the temperature of the test room. The wind velocity was also measured at this point. Further, the indoor air temperatures were also measured at different locations, as indicated by L_0_, L_1_, L_2_, L_3_ and L_4_, at a consistent height of 1.3 m. Correspondingly, the temperatures of the inner surface of the window inner layer and the outer surface of the window outer layer were measured, and their measuring point heights were the same as those of indoor air. The temperatures of the exterior wall and outdoor air were also measured. All temperatures were recorded using calibrated temperature sensors every 10 s. To prevent the interference of short-wave solar radiation and long-wave radiation from the environment, the temperature sensor probes were wrapped in tin foil. Table 2 presents the detailed specifications of instruments.

## 4 Results and analysis

**Fig. 5:**
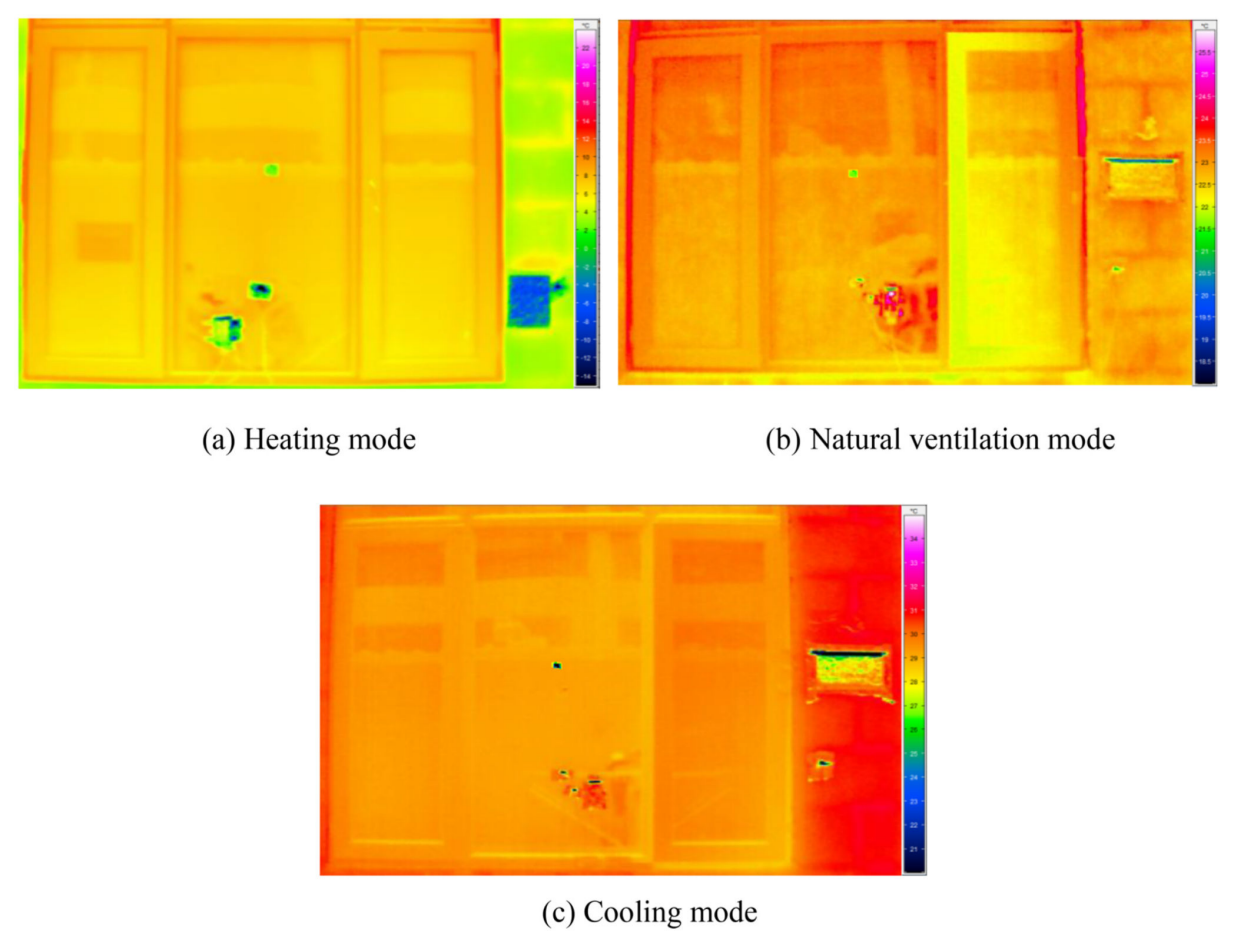
Full-size infrared images of the exterior window in (a) heating, (b) natural ventilation, and (c) cooling modes.

Full-size infrared images of the exterior window of the test room under heating, natural ventilation, and cooling modes are shown in Fig. 5. As shown in Fig. 5 (a), the infrared temperature of the window surface was significantly higher than that of the exterior wall in heating mode, which was the opposite of that in cooling mode (Fig. 5 (c)). In addition, the infrared temperature of the window surface was similar to that of the exterior wall in natural ventilation mode, as depicted in Fig. 5 (b). Owing to the high reflectivity of the tin foil, the temperature measuring points attached to the outer surface of the window appeared bright white. The average surface temperature within the 3 cm × 3 cm area near the actual measured point of the window glass was selected to represent the infrared temperature of the outer surface of the window outer layer. The infrared temperatures of the outer surface of the window outer layer and black tape were directly read by camera supporting software according to their corresponding emissivity and reflected ambient temperature. Since the infrared temperature of the black tape was equal to the infrared temperature of the external wall surface, the emissivity of the external wall surface was obtained. Then the infrared temperature of the exterior wall surface can be determined by entering its emissivity and reflected ambient temperature. Subsequently, the infrared temperature of the outer surface of the window outer layer was corrected based on the difference between the infrared temperature of the exterior wall surface and measured value. Lastly, the corrected infrared temperature of the outer surface of the window outer layer was substituted into the numerical model.

### 4.1 Model validation

#### 4.1.1 Validation of outer surface temperature of the window outer layer

**Fig. 6:**
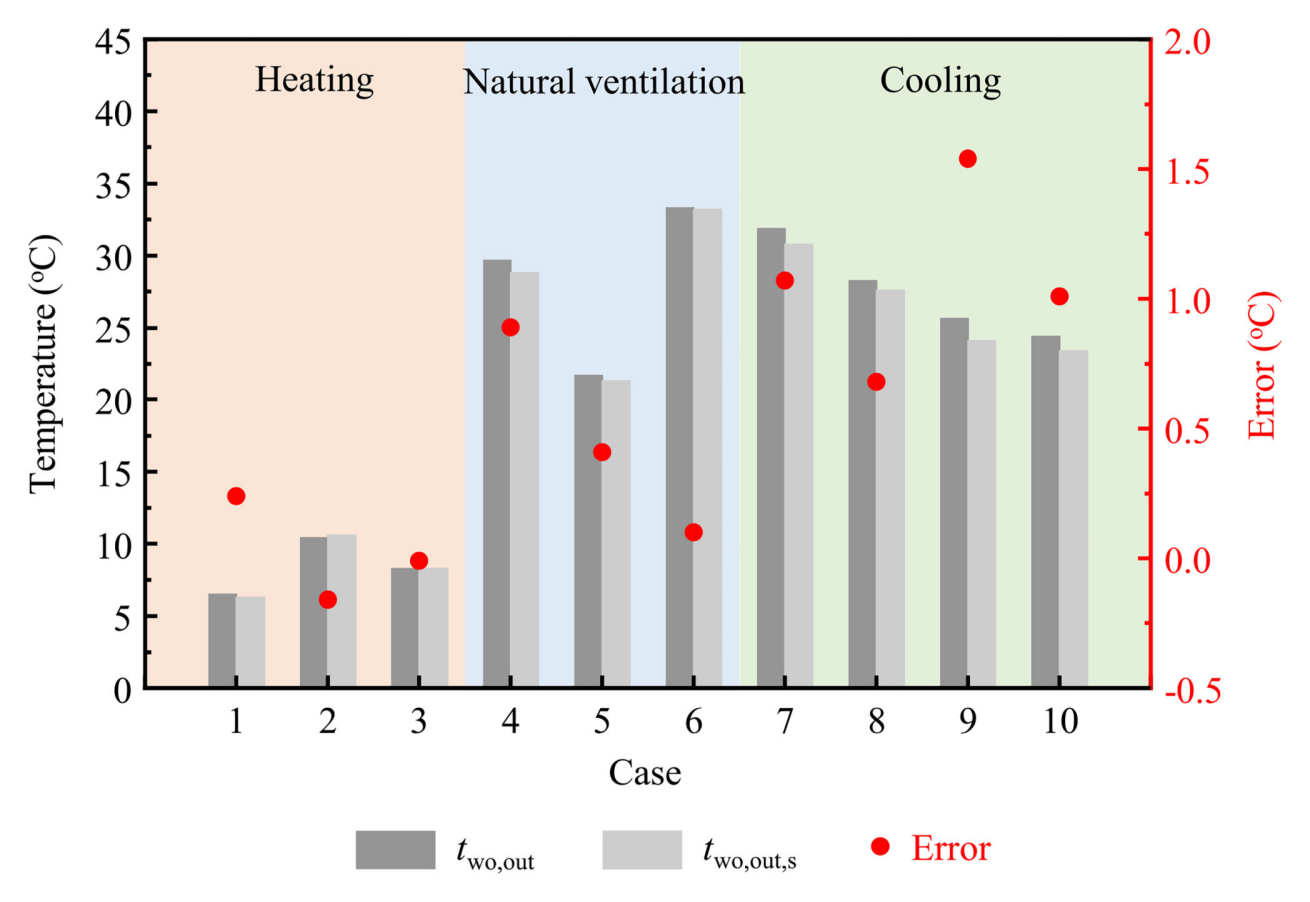
Comparisons of the infrared temperatures and measured data of the outer surface of the window outer layer in different modes. For case numbers refer to Table 1.

Comparisons of the infrared temperatures of the outer surface of the window outer layer and the measured data under different modes are presented in Fig. 6. The infrared temperatures of the outer surface of the window outer layer showed satisfactory agreement with the experimental data. In heating mode, the deviations of the outer surface of the window outer layer varied from −0.2 °C to 0.2 °C, and 0.1 °C–0.9 °C for natural ventilation mode. Correspondingly, the errors of the outer surface of the window outer layer were within the range of 0.7 °C–1.5 °C in cooling mode. This error mainly due to issues with the measuring instruments, as well as the comprehensive effects of building characteristics, climate conditions, and environmental defects.

#### 4.1.2 Validation of inner surface temperature of the window inner layer

**Fig. 7:**
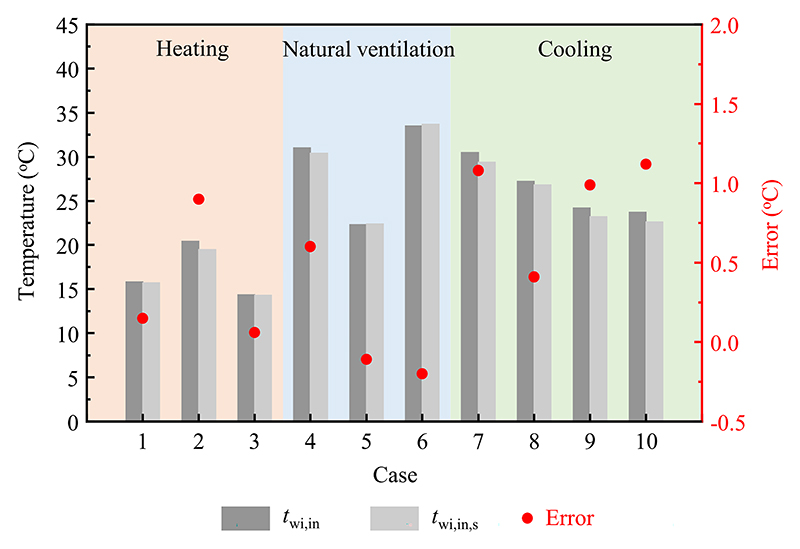
Comparisons of the estimated results and measured data of the inner surface temperature of the window inner layer in different modes. For case numbers refer to Table 1.

The estimated results of the inner surface temperature of the window inner layer obtained through the numerical model were validated with the experimental data under different modes. As shown in Fig. 7, the estimated results agreed well with the measured values. The errors of the inner surface temperature of the window inner layer were within the ranges of 0.1 °C–0.9 °C in heating mode and −0.2 °C–0.6 °C in natural ventilation mode. The deviations of the inner surface temperature of the window inner layer between estimated and measured data varied from 0.4 °C to 1.1 °C in cooling mode. All deviation values were acceptable, indicating the reliability of the established numerical model of the exterior window.

#### 4.1.3 Estimation of indoor air temperature of the test room

**Fig. 8:**
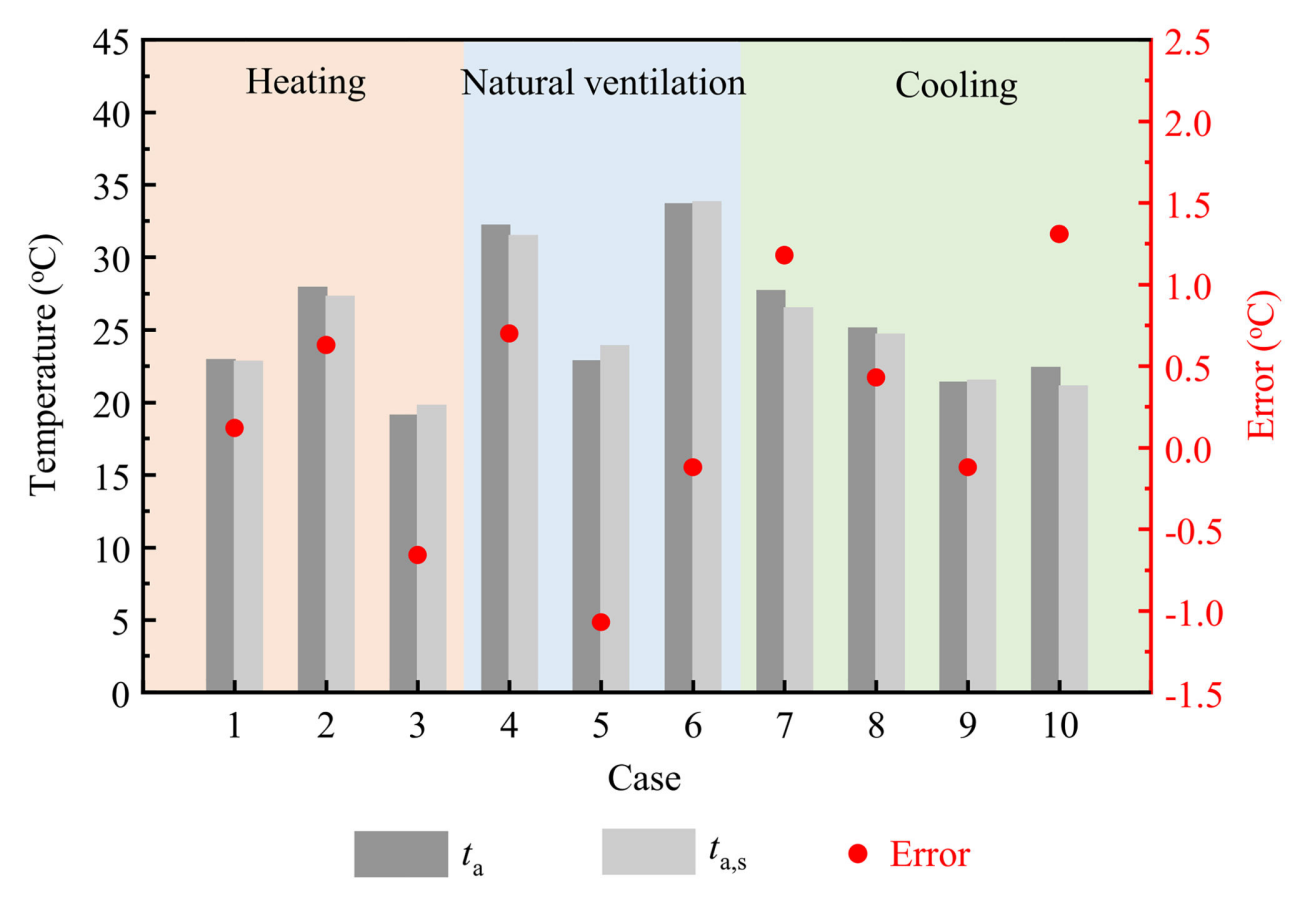
Comparisons of the estimated results and measured data of the indoor air temperature in different modes. For case numbers refer to Table 1.

The estimated results and experimental data for the indoor air temperature of the test room under different modes are compared in Fig. 8. The estimated results were in accordance with the measured values. In heating mode, the errors of the indoor air temperature varied from −0.7 °C to 0.6 °C. In natural ventilation mode, the deviations of the indoor air temperature were −1.1 °C–0.7 °C. In cooling mode, the errors of the indoor air temperature were within the range of −0.1 °C–1.3 °C. This was mainly due to the simplification of the numerical model and errors in the measuring instruments. The results indicated that the established numerical model can accurately predict the indoor air temperature of the test room.

### 4.2 Comparison with the window opening gap method

**Fig. 9:**
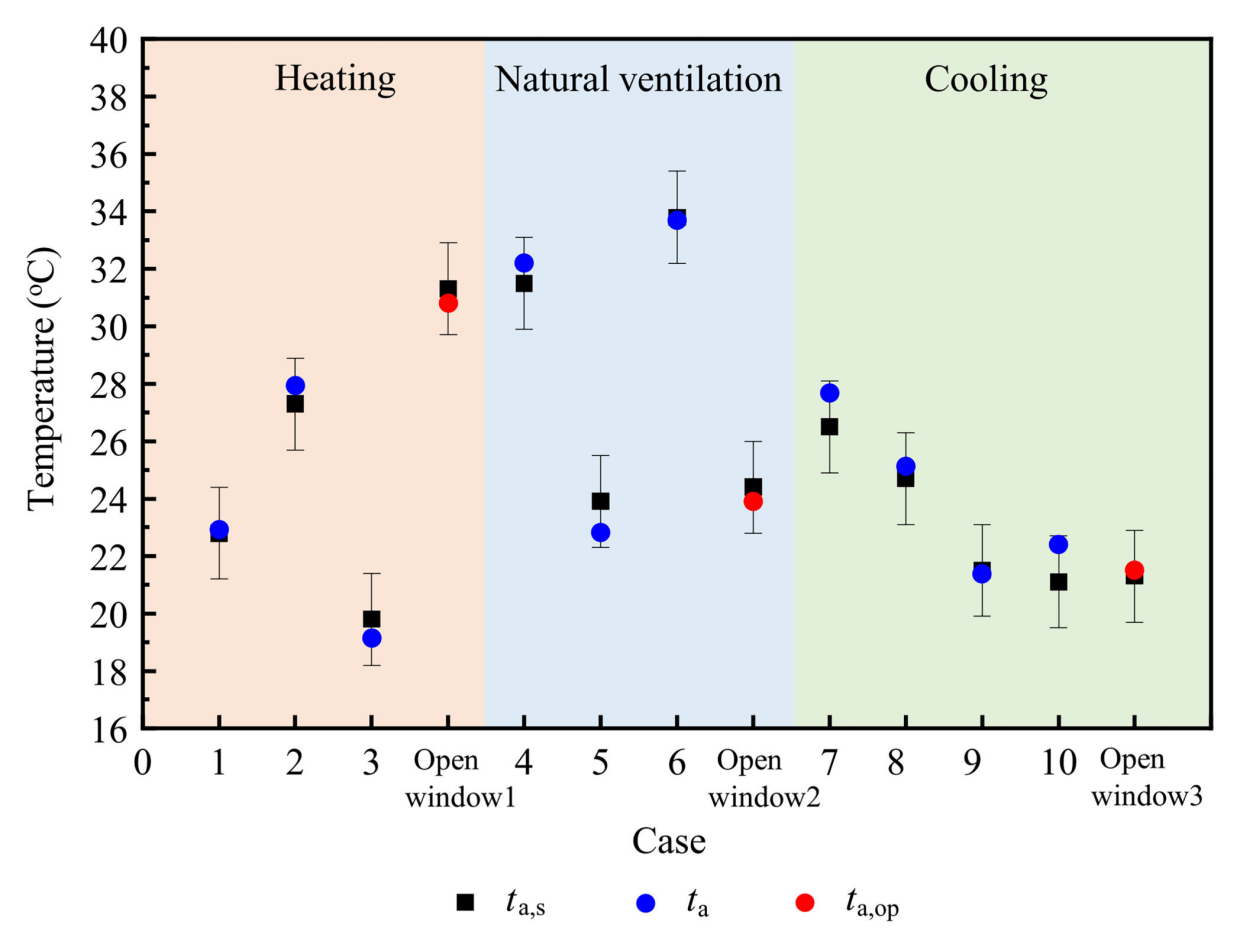
Comparisons of the results of indoor air temperatures under different methods. For case numbers refer to Table 1.

In a previous study, the window opening gap method was proposed to remotely estimate the indoor air temperature using an infrared camera, and the specific experimental scheme can be found in Ref. [32]. The results of calculating the indoor air temperature using the above method were compared with those obtained using the window opening gap method, as shown in Fig. 9. In heating mode, the error of the indoor air temperature through the window opening gap method was −0.5 °C, which was the same as that in natural ventilation mode. In cooling mode, the deviation of the indoor air temperature using the window opening gap method was 0.2 °C. The deviation values for the indoor air temperature were acceptable. In addition, obtaining the indoor air temperature through the window opening gap using an infrared camera is more convenient than calculating the indoor air temperature through the numerical model. Therefore, when the exterior window is opened, the window opening gap method should be adopted to predict the in-door air temperature. When the exterior window is closed, the numerical model calculation method can be used to evaluate the indoor air temperature in practical applications.

### 4.3 Influencing factors on indoor air temperature estimation

#### 4.3.1 Effect of the emissivity of the window surface

**Fig. 10:**
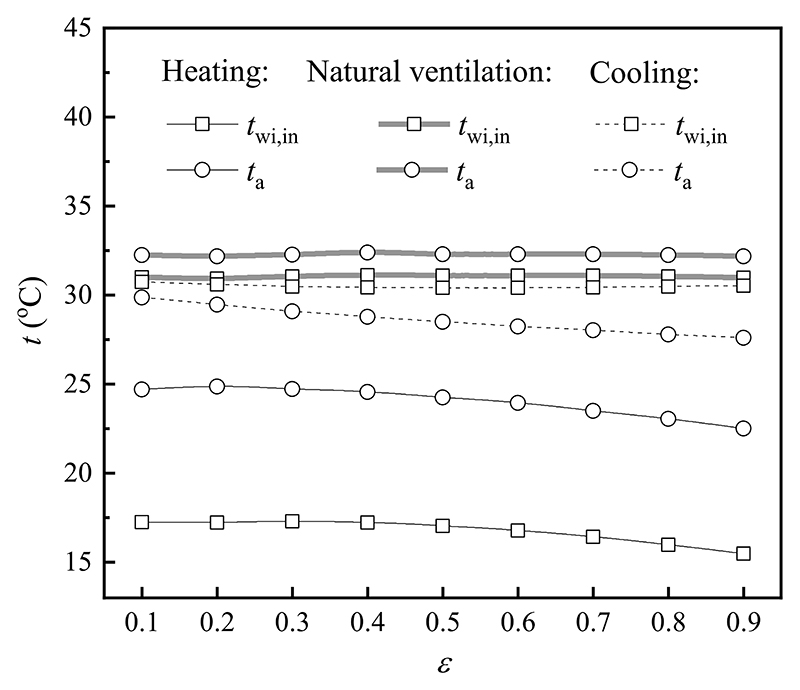
Effect of the emissivity of the window surface on various temperatures in different modes.

To investigate the effect of the emissivity of the window surface on the inner surface temperature of the window inner layer and indoor air temperature, the outdoor air temperature, the outer surface temperature of the window outer layer, and indoor and outdoor wind velocity were kept constant. Fig. 10 shows the variation trends in the inner surface temperature of the window inner layer and indoor air temperature with the emissivity of the window surface under different modes. Both the inner surface temperature of the window inner layer and indoor air temperature decreased slightly in heating and natural ventilation modes. When the emissivity of the window surface varied from 0.1 to 0.9, the indoor air temperature decreased by 2.2 °C and 0.1 °C, respectively. It was caused by the fact that as the emissivity of the window surface ascended, the total heat flux between the outer surface of the window outer layer and outdoor environment was increased, and then the inner surface temperature of the window outer layer was increased. However, the natural convection heat flux between the inner surface of the window outer layer and the outer surface of the window inner layer decreased owing to the increment of their radiation heat flux, and the inner and outer surface temperatures of the window inner layer decreased. Correspondingly, the natural convection heat flux between the inner surface of the window inner layer and indoor environment degraded, leading to the reduction of the indoor air temperature. In terms of the cooling mode, both the inner surface temperature of the window inner layer and indoor air temperature decreased with an increase in the emissivity of the window surface. The phenomenon can be explained using the same reason as that of heating and natural ventilation modes.

#### 4.3.2 Effect of the outdoor air temperature

**Fig. 11:**
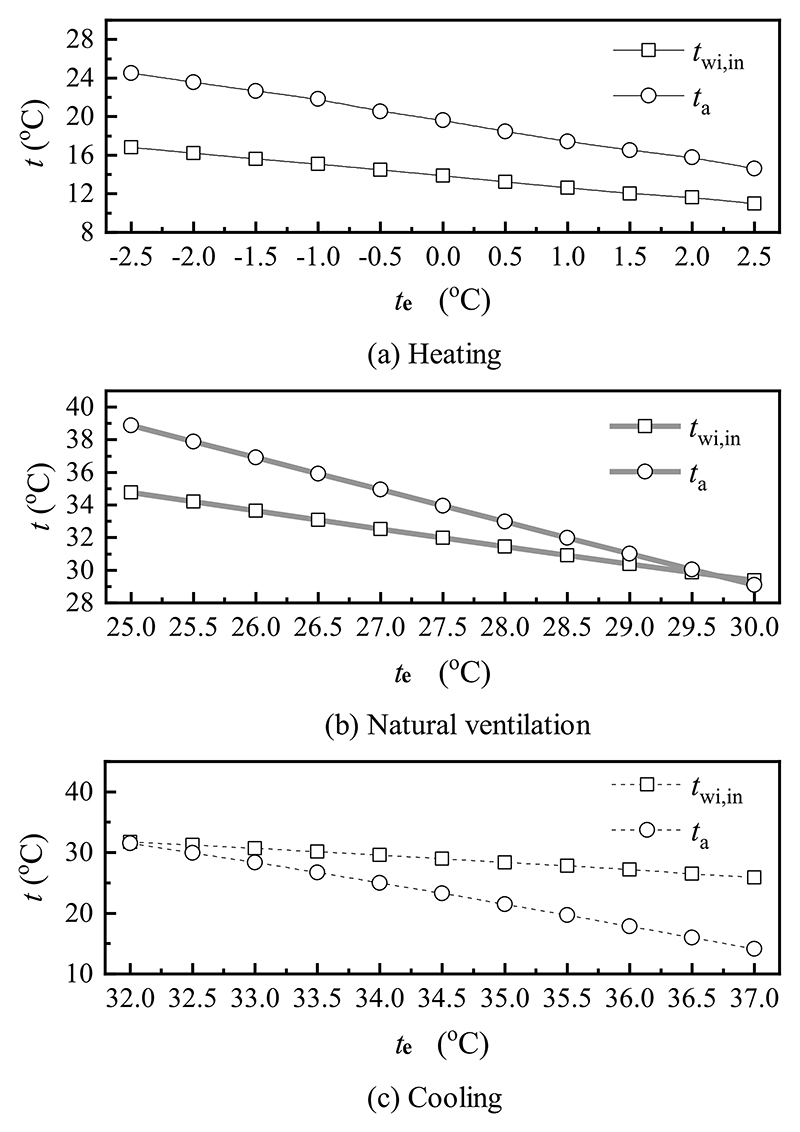
Effect of the outdoor air temperature on various temperatures in (a) heating, (b) natural ventilation, and (c) cooling modes.

When the emissivity of the window surface, the outer surface temperature of the window outer layer, and indoor and outdoor wind velocity remained unchanged, the effects of the outdoor air temperature on the inner surface temperature of the window inner layer and indoor air temperature under different modes are depicted in Fig. 11. As shown in Fig. 11 (a) and (b), with an increase in the outdoor air temperature, both the inner surface temperature of the window inner layer and indoor air temperature decreased. In heating mode, the inner surface temperature of the window inner layer and indoor air temperature reduced by 5.8 °C and 9.9 °C. In natural ventilation mode, both temperatures dropped by 5.4 °C and 9.8 °C. In addition, the indoor air temperature was consistently higher than that of the inner surface temperature of the window inner layer. This was likely because as the outdoor air temperature increased, the temperature difference between the outer surface temperature of the window outer layer and outdoor air temperature decreased, depressing the total heat flux and resulting in a reduction in the inner surface temperature of the window inner layer and indoor air temperature. As depicted in Fig. 11 (c), when the outdoor air temperature changed from 32.0 °C to 37.0 °C, the variations of the inner surface temperature of the window inner layer and indoor air temperature were within the ranges of 31.8 °C–25.9 °C and 31.5 °C–14.1 °C in cooling mode. In contrast, the indoor air temperature was lower than the inner surface temperature of the window inner layer continuously. The reason for this phenomenon was that the temperature difference between the outer surface temperature of the window outer layer and outdoor air temperature increased, improving the total heat flux and leading to a decrease in the inner surface temperature of the window inner layer and indoor air temperature.

#### 4.3.3 Effect of the outer surface temperature of the window outer layer

**Fig. 12:**
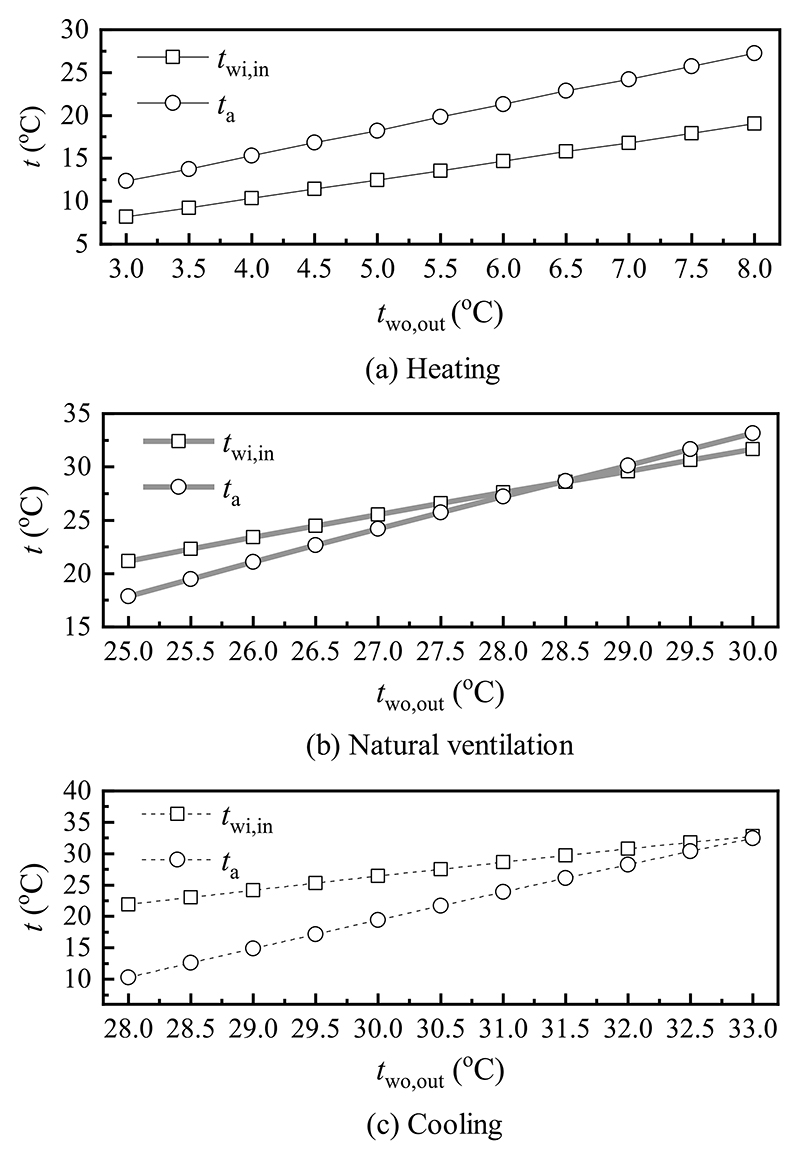
Effect of the outer surface temperature of the window outer layer on various temperatures in (a) heating, (b) natural ventilation, and (c) cooling modes.

When the emissivity of the window surface, outdoor air temperature, and indoor and outdoor wind velocity were kept constant, the variation trends of the inner surface temperature of the window inner layer and indoor air temperature with the outer surface temperature of the window outer layer under different modes are displayed in Fig. 12. As shown in Fig. 12 (a) and (b), with an increase in the outer surface temperature of the window outer layer, both the inner surface temperature of the window inner layer and indoor air temperature increased. In heating mode, the inner surface temperature of the window inner layer and indoor air temperature rose by 10.8 °C and 14.9 °C. In natural ventilation mode, both temperatures increased by 10.5 °C and 15.3 °C. It can be explained by the increased difference between the outer surface temperature of the window outer layer and outdoor air temperature, which enhanced the natural convective and radiation heat transfer between them, thereby increasing the total heat flux and raising the inner surface temperature of the window inner layer and indoor air temperature. Fig. 12 (c) indicated that the inner surface temperature of the window inner layer and indoor air temperature were positively proportional to the outer surface temperature of the window outer layer in cooling mode. When the outer surface temperature of window outer layer varied from 28.0 °C to 33.0 °C, the inner surface temperature of the window inner layer and indoor air temperature were within the ranges of 21.9 °C–32.8 °C and 10.3 °C–32.4 °C. This behavior can be attributed that with the rise of the outer surface temperature of the window outer layer, the temperature difference between the outer surface temperature of the window outer layer and outdoor air temperature degraded, then the natural convective and radiation heat transfer between them were reduced. This leads to a decrease in the total heat flux and an increase in the inner surface temperature of the window inner layer and indoor air temperature.

#### 4.3.4 Effect of the outdoor wind velocity

**Fig. 13:**
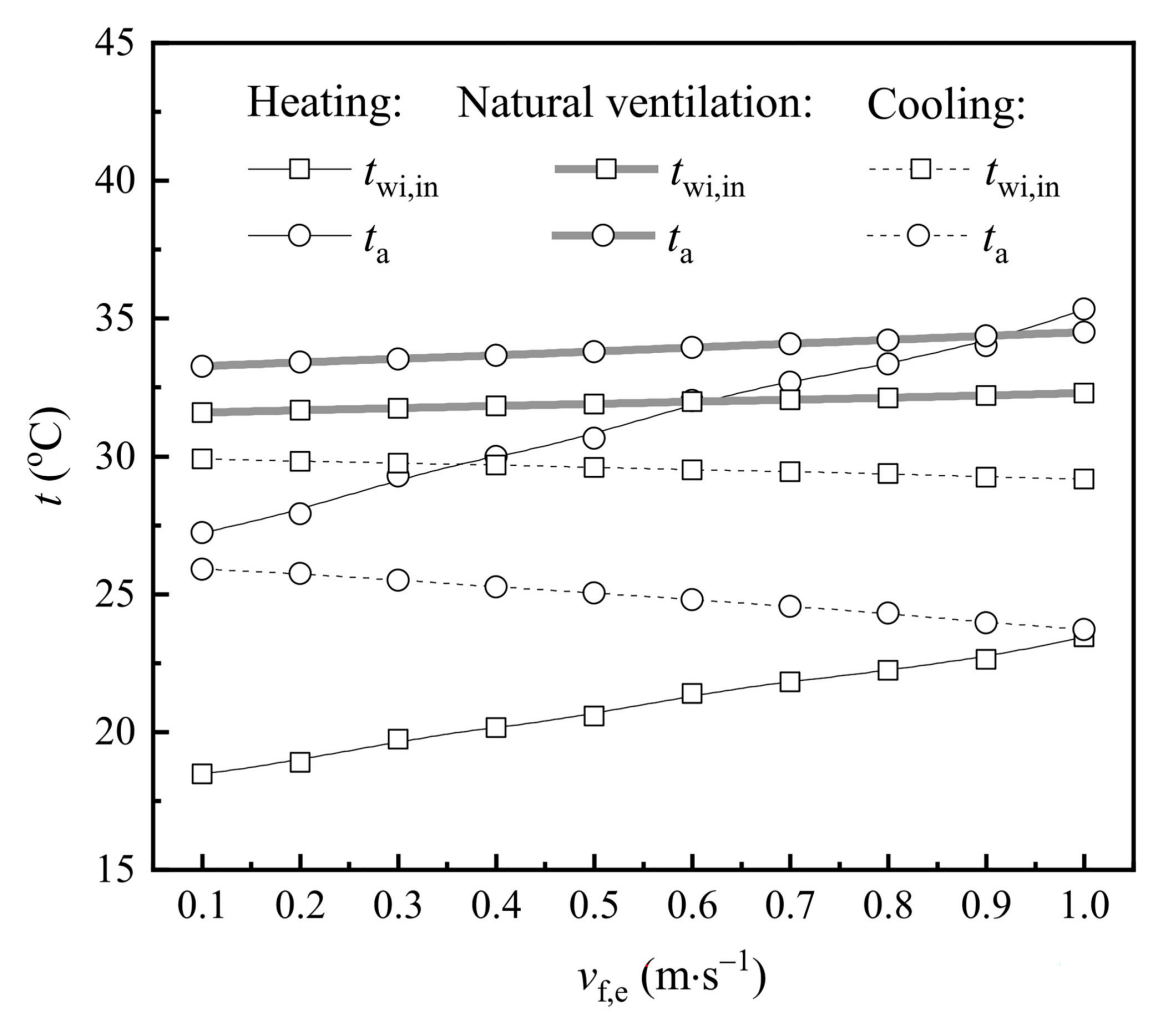
Effect of the outdoor wind velocity on various temperatures in different modes.

Fig. 13 presents the changes of the inner surface temperature of the window inner layer and indoor air temperature with different outdoor wind velocities, given that the emissivity of the window surface, the outer surface temperature of the window outer layer, outdoor air temperature, and indoor wind velocity were fixed. With an increase in the outdoor wind velocity, both the inner surface temperature of the window inner layer and indoor air temperature ascended. When the outdoor wind velocity varied from 0.1 to 1.0 m s^-1^, the inner surface temperature of the window inner layer and indoor air temperature increased by 5.0 °C and 8.1 °C in heating mode. Correspondingly, both temperatures increased by 0.7 °C and 1.2 °C in natural ventilation mode. It can be attributed that the increased convective heat transfer intensity between the outer surface of the window outer layer and outdoor air, which enhanced the total heat flux, leading to the increment of the inner surface temperature of the window inner layer and indoor air temperature. With regard to the cooling mode, the inner surface temperature of the window inner layer and indoor air temperature reduced by 0.7 °C and 2.2 °C with an increase in the outdoor wind velocity. The main reason for this phenomenon was similar to that of heating and natural ventilation modes.

#### 4.3.5 Effect of the indoor wind velocity

**Fig. 14:**
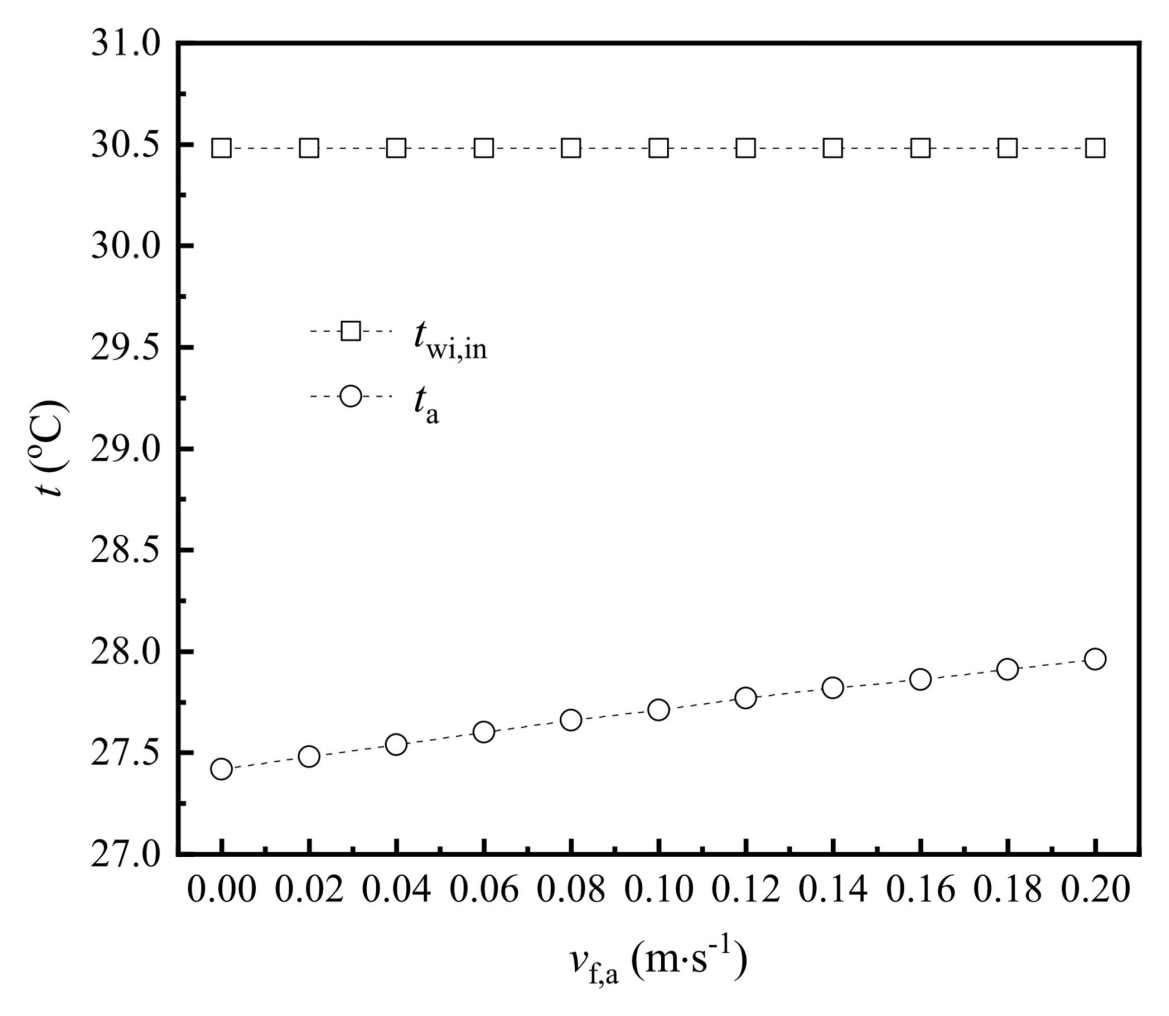
Effect of the indoor wind velocity on various temperatures in cooling mode.

To evaluate the inner surface temperature of the window inner layer and indoor air temperature under different indoor wind velocities in cooling mode, the emissivity of the window surface, outdoor air temperature and wind velocity, and the outer surface temperature of the window outer layer were maintained at 0.837, 33.2 °C, 0.1 m s^−1^ and 31.9 °C, respectively. Fig. 14 depicts the variation trends of the inner surface temperature of the window inner layer and indoor air temperature relative to the indoor wind velocity. The inner surface temperature of the window inner layer remained constant at 30.5 °C with the rise of the indoor wind velocity. However, the indoor air temperature ascended linearly, within the range of 27.4 °C–28.0 °C. This pattern can be attributed that the convective heat transfer coefficient between the inner surface of the window inner layer and indoor environment enhanced, whereas the total heat flux was fixed, resulting in an increase in the indoor air temperature.

#### 4.3.6 Sensitivity analysis of influencing factors

**Fig. 15:**
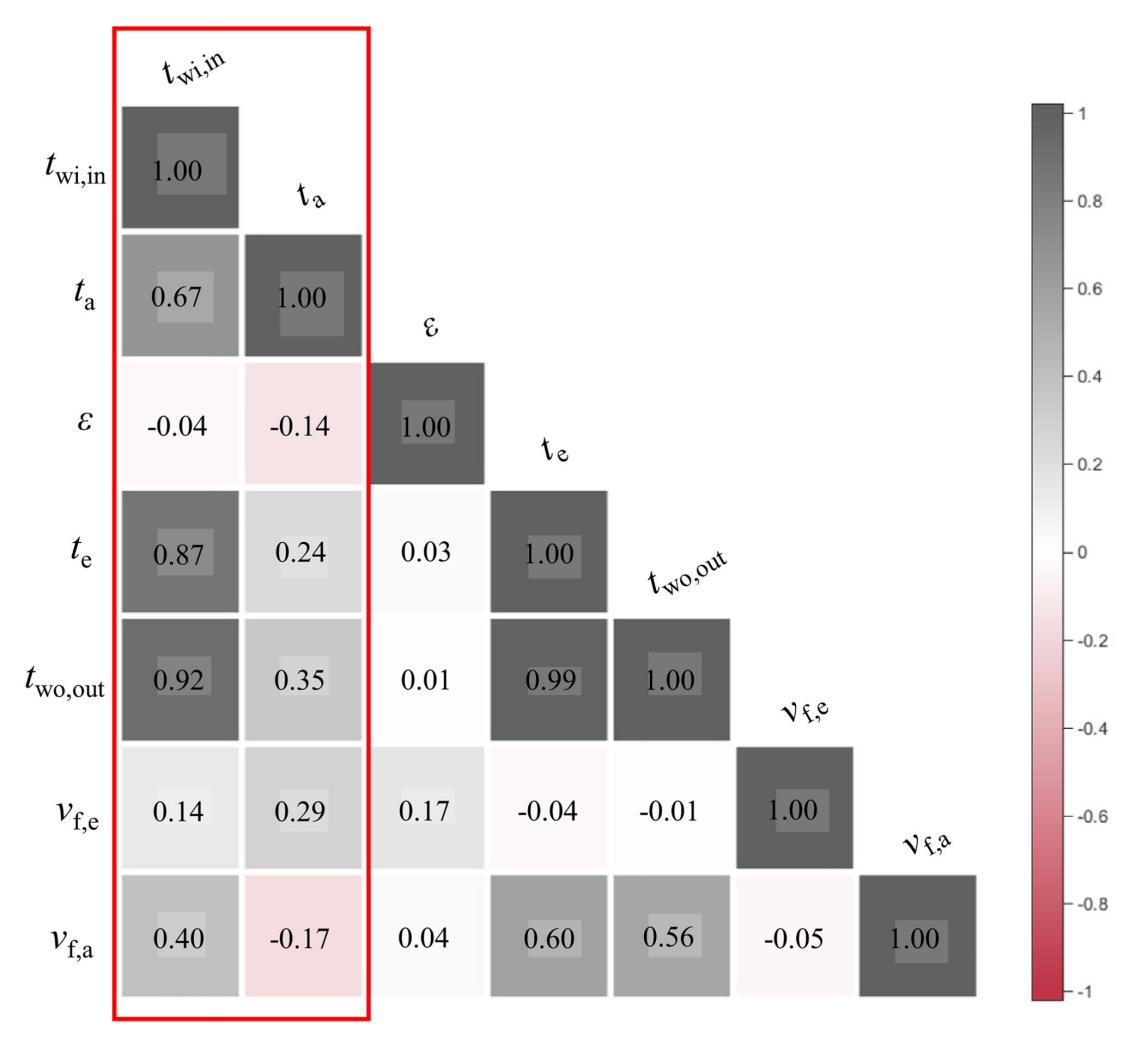
The correlation degrees among different influencing factors.

The sensitivity analysis among different influencing factors and the inner surface temperature of the window inner layer as well as indoor air temperature were conducted by SPSS software. The calculation results are shown in Fig. 15. In terms of the inner surface temperature of the window inner layer, the crucial factor was the outer surface temperature of the window outer layer with the correlation degree of 0.92. The correlation degree of these influencing factors was ordered as: *t*_wo,out_ > *t*_e_ > *v*_f,a_ > *v*_f,e_ > *ε*, indicating that the outer surface temperature of the window outer layer was imperative for estimating the inner surface temperature of the window inner layer. In terms of the indoor air temperature, the outer surface temperature of the window outer layer was also a critical factor and its correlation degree was 0.35. The correlation degree of these influencing factors was sorted as: *t*_wo,out_ > *v*_f,e_ > *t*_e_ > *v*_f,a_ > *ε*. It can be denoted that the outer surface temperature of the window outer layer played a prominent role for predicting the indoor air temperature. Therefore, it is important to pay attention to the measurement accuracy of the outer surface temperature of the window outer layer, which is helpful to estimate the indoor air temperature from outside without intrusion using infrared technology in practical applications.

## 5 Discussion

The present study indicates the preliminary feasibility of using infrared technology to estimate the indoor air temperature from outside without intrusion. However, the current results were obtained based on controlled laboratory setting, and could not be extended to actual onsite conditions without further tests. The existing method is only tested for applications in buildings located on the first floor. Going forward, the reliability and applicability of this method in more complicated conditions should be investigated. The effect of the infrared camera distance, observation angle and shading device in the window should also be explored. In addition, the potential influence of humidity level and condensation and frozen water on window glass surface also need to be studied. Moreover, this method currently does not consider the stratification of indoor air temperature. If the indoor air temperature becomes non-uniform, the feasibility of this method should also be investigated in future work.

## 6 Conclusions

In this study, an exploratory approach was proposed to estimate the indoor air temperature by obtaining the building window surface temperature from outside using infrared technology. A numerical model between the indoor and outdoor air through window glass was developed. The estimated indoor air temperatures were validated with the measured data under different modes. Based on the numerical model, the effects of the window property parameters and ambient parameters on indoor air temperature estimation were studied. The main conclusions were as follows.

(1) The established numerical model between the indoor and outdoor air through window glass was credible, and the estimated results fit reasonably well with the measured data. The deviations of the indoor air temperature in heating, natural ventilation and cooling modes were within the ranges of −0.7 °C–0.6 °C, −1.1 °C–0.7 °C and −0.1 °C–1.3 °C, respectively.
(2) When the exterior window is opened, the window opening gap method is suitable to evaluate the indoor air temperature. When the exterior window is closed, the proposed approach is more capable of estimating the indoor air temperature.
(3) The current results are limited to the controlled laboratory setting, which could differ from actual onsite conditions. Further studies are necessary to test the feasibility of this approach in more complicated scenarios.

## Figures and Tables

**Table 1:** The conditions and environmental parameters in the test room.

Case	Experimental condition	*t*_*r*_ (°C)	*t*_ex,s_ (°C)	*t*_e_ (°C)	*t*_wo,out,s_ (°C)	*t*_wi,in,s_ (°C)	*t*_a,s_ (°C)	*v*_f,a_ (m⋅s^−1^)
1	Heating	−8.0	2.4	−1.7	7.9	15.7	22.9	–
2	Heating	−5.2	6.3	1.6	10.6	17.1	27.3	–
3	Heating	−4.5	5.9	2.8	8.3	12.6	14.3	–
4	Natural ventilation	22.0	30.3	28.4	28.8	30.4	31.5	–
5	Natural ventilation	22.6	21.5	21.1	21.3	22.4	23.9	–
6	Natural ventilation	28.2	34.0	33.1	33.2	33.7	33.8	–
7	Cooling	31.3	33.8	33.2	30.8	29.4	26.5	0.1
8	Cooling	28.3	29.6	29.3	27.6	26.8	24.7	0.1
9	Cooling	24.2	26.8	27.0	24.1	23.2	21.5	0.1
10	Cooling	25.0	25.8	25.1	23.4	22.6	21.1	0.1

**Table 2:** Detailed specifications of instruments.

Measured parameter	Instrument name	Instrument number	Manufacturer	Country	Full scale	Accuracy
Temperature	Temperature sensor	WZY-1	Beijing Tianjian Huayi Technology Development Co., Ltd	China	−20 -80 °C	±0.3 °C
Infrared image	Infrared thermal imager	VarioCAM	Jenoptik	Germany	−40 -1200 °C	±2.0%
Wind velocity	Anemometer	FB-1	Beijing Tianjian Huayi Technology Development Co., Ltd	China	0−30 m s^−1^	±5.0%

## Data Availability

Data will be made available on request.

## Nomenclature

A: Control volume area (m^2^)
*Gr*_H_: Grashof number
*g*: Gravitational acceleration (m⋅s^−2^)
*H*: Window height (m)
*h*: Convective heat transfer coefficient (W⋅m^−2^⋅K^−1^)
*Nu*_f_: Nusselt number
*Pr*: Prandtl number
*Q*: Heat exchange capacity (W)
*t*: Temperature (°C)
*v*_f_: Wind velocity (m⋅s^−1^)
*Δt*: Temperature difference (°C)

## Greek symbols

*λ*: Thermal conductivity (W⋅m^−1^⋅K^−1^)
*δ*: Thickness (m)
ε: Emissivity
*σ*: Stefan-Boltzmann constant (5.67 × 10^−8^ W m^−2^ K^−4^)
*α*: Volume expansion coefficient (1 K^−1^)
*ν*: Kinematic viscosity (m^2^⋅s)

## Subscripts

a: indoor air
b: glass sandwich intersurface
e: outdoor air
ex: exterior wall
in: inner surface
op: open window
out: outer surface
r: reflection
rad: radiation
s: sensor
w: window glass
wi: window inner layer
wo: window outer layer

## Abbreviations

IR: infrared thermography
